# Supplementary material for: Associations of psychiatric disease and ageing with FKBP5 expression converge on superficial layer neurons of the neocortex
Source: Acta Neuropathol. 2023 Feb 2;145(4):439–59. doi: 10.1007/s00401-023-02541-9 (PMC10020280; doi:10.1007/s00401-023-02541-9)
Supplement: Supplementary file 1 — Supplementary file1 (DOCX 8786 KB) [file 401_2023_2541_MOESM1_ESM.docx]

Supplementary Information

**Associations of psychiatric disease and aging on *FKBP5* expression converge on superficial layer neurons of the neocortex**

Natalie Matosin^1,2,3^*, Janine Arloth^1,4^, Darina Czamara^1^, Katrina Z. Edmond^2,3^, Malosree Maitra^6^, Anna Sophie Fröhlich^1,5^, Silvia Martinelli^1,5^, Dominic Kaul^2,3^, Rachael Bartlett^2,3^, Amber R. Curry^2,3^, Nils C. Gassen^1,8^, Kathrin Hafner^1^, Nikola S Müller^4^, Karolina Worf^4^, Ghalia Rehawi^1,4^, Corina Nagy^6,9^, Thorhildur Halldorsdottir^7^, Cristiana Cruceanu^1^, Miriam Gagliardi^10^, Nathalie Gerstner^1,4,5^, Maik Ködel^1^, Vanessa Murek^1,10^, Michael J Ziller^1,10^, Elizabeth Scarr^11^, Ran Tao^12^, Andrew E. Jaffe^12^, Thomas Arzberger^13,14^, Peter Falkai^1,13^, Joel E. Kleinmann^12,15^, Daniel R. Weinberger^12,15^, Naguib Mechawar^6,9^, Andrea Schmitt^13,16^, Brian Dean^11^, Gustavo Turecki^6,9,18^, Thomas M. Hyde^12,15^, Elisabeth B. Binder^1,19^*

^1^ Department of Translational Research in Psychiatry, Max-Planck Institute of Psychiatry, Munich, Germany

^2^ Illawarra Health and Medical Research Institute, Northfields Ave, Wollongong 2522, Australia

^3^ Molecular Horizons, School of Chemistry and Molecular Biosciences, Faculty of Science, Medicine and Health, University of Wollongong, Northfields Ave, Wollongong 2522, Australia

^4^ Institute of Computational Biology, Helmholtz Zentrum München, Neuherberg 85764, Germany

^5^ International Max Planck Research School for Translational Psychiatry, Munich, Germany

^6^ McGill Group for Suicide Studies, Douglas Mental Health University Institute, Montreal, Quebec, Canada

^7^ Department of Psychology, Reykjavik University, Reykjavik, Iceland

^8^ Neurohomeostasis Research Group, Institute of Psychiatry, University of Bonn, Clinical Centre, Bonn, Germany

^9^ Department of Psychiatry, McGill University, Montreal, Quebec, Canada

^10^Department of Psychiatry, University of Münster, Münster, Germany

^11^ Melbourne Veterinary School, Faculty of Veterinary and Agricultural Sciences, The University of Melbourne, Parkville, Victoria 3010

^12^ The Lieber Institute for Brain Development, Johns Hopkins University Medical Campus, Baltimore, MD, USA

^13^ Department of Psychiatry and Psychotherapy, University Hospital, Ludwig-Maximilians University Munich, Nussbaumstrasse 7, 80336 Munich, Germany

^14^ Centre for Neuropathology and Prion Research, Ludwig-Maximilians University Munich, Nussbaumstrasse 7, 80336 Munich, Germany

^15^ Department of Psychiatry and Behavioral Sciences at Johns Hopkins University School of Medicine

^16^ Laboratory of Neuroscience (LIM27), Institute of Psychiatry, University of Sao Paulo, Rua Dr. Ovidio Pires de Campos 785, 05453-010 São Paulo, Brazil

^17^ Synaptic Neurobiology and Cognition Laboratory, Florey Institute for Neuroscience and Mental Health, Parkville, VIC, Australia

^18^ Department of Human Genetics, McGill University, Montreal, Quebec, Canada

^19^ Department of Psychiatry and Behavioral Sciences, Emory University School of Medicine, Atlanta, USA

*** Correspondence**

Dr Natalie Matosin

University of Wollongong, Wollongong Australia

[nmatosin@uow.edu.au](mailto:nmatosin@uow.edu.au)

+61 2 4221 5150

Prof Elisabeth Binder

Max Planck Institute of Psychiatry, Munich Germany

[binder@psych.mpg.de](mailto:binder@psych.mpg.de)

+49 89 30622 586

Contents

1. **Extended Methods**
2. Dissection protocols, clinical and demographic details for postmortem cohorts
   1. Supplementary Table 1: Demographics of the LIBD lifetime cohort
   2. Supplementary Table 2: Demographics of the Victorian Brain Bank cohort
   3. Supplementary Table 3: Demographics from the Munich Brain Bank cohort
   4. Supplementary Table 4: Demographics from the Stanley Neuropathology Consortium
   5. Supplementary Table 5: Demographics from the NSW Tissue Resource Centre cohort
   6. Supplementary Table 6: Demographics from the Douglas-Bell Canada Brain Bank cohort
3. Primer design for real-time quantitative PCR experiments
   1. Supplementary Table 7: RT-qPCR primer design
4. Antibody validation experiments
   1. Supplementary Table 8: FKBP51 antibodies validated in FKBP51 knock-out cells
   2. Supplementary Figure 1: Validation of FKBP51 antibodies in *FKBP5* knock-out HELA cells using immunoblot
   3. Supplementary Table 9: Summary of antibodies used for immunohistochemistry experiments
5. Fluorescence immunohistochemistry method and quantification
6. Golgi-Cox staining method and quantification
7. **Extended Results**

- Supplementary Table 10: Further information about statistical methods used.
- Supplementary Table 11: Sample sizes for genotype analyses
- Supplementary Figure 2: Association of *FKBP5/*1 expression with age in Cohort 2, 3 and 4
- Supplementary Table 12: Schizophrenia-control differences in *FKBP5* gene expression between the major cell-types and excitatory neuron sub-clusters from Cohort 5 using linear regression modelling
- Supplementary Table 13: Depression differences in *FKBP5* gene expression between the major cell-type clusters and excitatory neuron sub-clusters from Cohort 6 using linear regression modelling
- Supplementary Figure 3: Case-control differences in *FKBP5* gene expression in each cell-type cluster from Cohort 5 and Cohort 6
- Supplementary Table 14: Results of Spearman correlations assessing the relationship between *FKBP5* gene expression with age in each cell cluster from Cohort 5
- Supplementary Table 15: Results of Spearman correlations assessing the relationship between *FKBP5* gene expression with age in each cell cluster from Cohort 6
- Supplementary Table 16: Results of Spearman correlations in Cohort 4, assessing the relationship between age and (a) FKBP51 staining intensity on NeuN+ neurons, and (b) the number of FKBP51+ NeuN+ cells in the ACC.
- Supplementary Figure 4. Dimensionality reduction uniform manifold approximation and projection (UMAP) plots depicting (a) *BDNF* expression across cell clusters and (b) bar plot showing average *FKBP5* gene expression per cell-type cluster from Cohort 5.

1. **References**

**A. EXTENDED METHODS**

**1. Dissection protocols, clinical and demographic details for postmortem cohorts**

*Postmortem brain Cohort 1*

The LIBD cohort (Cohort 1; Supplementary Table 1) consisted of 252 control subjects ranging from neonate, infant, child, adolescent, and adult age-groups, and 184 schizophrenia (SZ) subjects, 69 bipolar disorder (BPD) and 152 major depressive disorder (MDD) cases. Brains from Cohort 1 were collected at the Clinical Brain Disorders Branch (CBDB), the US National Institute of Mental Health (NIMH), the Northern Virginia and the District of Columbia Medical Examiner’s Office (informed consent obtained from legal next of kin for all cases; NIMH Protocol 90-M-0142). Additional fetal, child, and adolescent brain samples were obtained through the National Institute of Child Health and Human Development Brain and Tissue Bank for Developmental Disorders (N01-HD-4-3368 and N01-HD-4_3383). Briefly, DLPFC grey matter from fetal cases was extracted using a dental drill from hemisected 1-1.5cm coronal slabs. For non-fetal cases, Brodmann area 9 was dissected from the middle frontal gyrus immediately anterior to the genu of the corpus callosum. Dissected tissues were pulverized and stored at -80°C. Detailed information about brain tissue collection and retrieval are available elsewhere (Tao, Davis et al. 2017).

**Supplementary Table 1**. Demographics of the LIBD lifetime cohort

|  | **Group; mean (range)*** | | | |
| --- | --- | --- | --- | --- |
| **Descriptive** | **CTRL** | **MDD** | **BD** | **SCZ** |
| Subjects | 340 | 132 | 59 | 102 |
| Age at death, yr | 30.12 (14 weeks prenatal to 85) | 43.7 (21-64) | 43.7 (21-65) | 46.0 (20-62) |
| Sex, male:female | 228:112 | 79:53 | 32:27 | 69:33 |
| PMI, h | 24.79 (1-90) | 36.4 (5-160) | 29.5 (5-65) | 39.0 (7-142) |
| pH | 5.72 (5.9-7.1) | 6.4 (5.9-7.3) | 6.3 (5.9-6.9) | 6.4 (5.9-7.0) |
| RIN | 8.4 (5-10) | 8.0 (5.1-9.5) | 7.8 (5.0-9.3) | 8.0 (5.4-9.4) |
| Cause of Death^†^, 1:2:3:4:5 | 2:53:186:0:37 | 2:20:28:79:1 | 2:7:11:39:0 | 3:15:64:20:0 |
| Ethnicity^‡^ 1:2:3:4 | 145:177:9:9 | 116:11:3:2 | 49:5:2:3 | 55:42:2:3 |

***Abbreviations:*** CTRL, control; MDD, major depressive disorder; BPD, bipolar disorder; SCZ, schizophrenia; PMI, postmortem interval; RIN, RNA integrity number. ^*^Unless otherwise indicated. ^†^ 1 = Undetermined, 2 = Accident, 3 = Natural, 4 = Suicide, 5 = Homicide. ‡ 1 = Caucasian, 2 = African American, 3 = Hispanic, 4 = Asian.

*Postmortem brain Cohort 2*

The Victorian brain bank cohort (Cohort 2; Supplementary Table 2) consists of 20 schizophrenia, 20 major depression and 16 bipolar subjects, as well as 20 matched controls. The Diagnostic Instrument for Brain Studies was used to conduct case history reviews to reach a diagnostic consensus using the DSM-IV criteria. Regarding dissection, briefly, BA9 was isolated from the lateral surface of the frontal lobe containing the middle frontal gyrus superior to the inferior frontal sulcus. Tissue blocks were stored at -80°C until processing for downstream applications. The collection and dissection process has been previously described in detail (Scarr, Udawela et al. 2018).

**Supplementary Table 2**: Demographics of the Victorian Brain Bank cohort, postmortem samples of dorsolateral prefrontal cortex (BA9) from the left hemisphere

|  | **Group; mean (range)*** | | | |
| --- | --- | --- | --- | --- |
| **Descriptive** | **CTRL** | **MDD** | **BPD** | **SCZ** |
| Subjects | 20 | 20 | 16 | 20 |
| Age at death (years) | 59.2 (32-80) | 85.0 (27-87) | 59.4 (31-79) | 56.4 (30-82) |
| Sex (male:female) | 11:9 | 11:9 | 8:8 | 11:9 |
| Suicide (no:yes) | 19:1 | 3:17 | 10:6 | 14:6 |
| Brain pH | 6.3 (5.9-6.7) | 6.5 (5.6-6.9) | 6.3 (6.0-6.5) | 6.3 (5.5-6.6) |
| PMI (hours) | 43.0 (17-72) | 43.4 (11-72) | 38.7 (8-63) | 44.2 (20-66) |
| Brain weight (grams) | 1354 (1140-1655) | 1285 (1030-1573) | 1244 (952-1630) | 1337 (1110-1659) |

***Abbreviations:*** CTRL, control; MDD, major depressive disorder; BPD, bipolar disorder; SCZ, schizophrenia; PMI, postmortem interval; RIN, RNA integrity number. ^*^Unless otherwise indicated

*Postmortem brain Cohort 3*

The Munich Neurobiobank cohort (Cohort 3; Supplementary Table 1b) consists of 24 control subjects with ages evenly distributed over adulthood, from 37 to 88 years of age. Clinical records were provided by relatives and general practitioners and all assessments and postmortem evaluations were conducted in accordance with the Ethics Committee of the Faculty of Medicine, University of Heidelberg, Germany. Controls had no history of alcohol or drug abuse, severe physical or psychiatric illness. Tissue blocks (~1-1.5cm^2^) were dissected from the anterior-most point of the superior frontal gyrus, corresponding to BA9. Braak neuropathological examinations were staged at ≤2 for all subjects. Tissue blocks were stored at -80°C until processing for downstream applications.

**Supplementary Table 3**: Demographics from Munich Brain Bank, postmortem control cohort with samples from the dorsolateral prefrontal cortex (BA9)

|  | **Group; mean (range)*** |
| --- | --- |
| **Descriptive** | **CTRL** |
| Subjects | 24 |
| Age at death (years) | 65.5 (37-88) |
| Sex (male:female) | 13:11 |
| Hemisphere (left:right) | 11:13 |
| PMI (hours) | 32.6 (13-71) |
| RIN | 6.1 (3.0-8.3) |

***Abbreviations:*** CTRL, control; MDD, major depressive disorder; BPD, bipolar disorder; SCZ, schizophrenia; PMI, postmortem interval; RIN, RNA integrity number. ^*^Unless otherwise indicated

*Postmortem brain Cohort 4*

The Stanley Neuropathology Consortium (Cohort 4, Supplementary Table 4) consists of 60 adult subjects (schizophrenia, major depression, bipolar disorder and matched controls, n=15/group). Tissue was isolated from the ventral portion of BA24, anterior to the mid-level of the corpus callosum. This area was chosen due to its direct projections the BA9/11 regions. The samples were matched for age, sex, race, and PMI. Detailed clinical and demographic information regarding these cohorts have been published previously (Torrey, Webster et al. 2000).

**Supplementary Table 4**: Demographics from the Stanley Neuropathology consortium, postmortem control cohort with samples from the anterior cingulate cortex (BA24)

|  | **Group; mean (range)*** | | | |
| --- | --- | --- | --- | --- |
| **Descriptive** | **CTRL** | **MDD** | **BPD** | **SCZ** |
| Subjects | 15 | 15 | 15 | 15 |
| Age at death (years) | 48.1 (29-68) | 46.5 (30-65) | 42.3 (25-61) | 44.2 (25-62) |
| Age of onset | - | 33.9 (11-54) | 21.5 (7-39) | 23.2 (13-42) |
| Sex (male:female) | 9M, 6F | 9M, 6F | 9M, 6F | 9M, 6F |
| PMI (hours) | 23.7 (8-42) | 27.5 (7-47) | 32.5 (13-62) | 33.7 (12-61) |
| FST (days) | 338.27 (31-774) | 434 (86-931) | 620 (224-836) | 621.13 (68-938) |
| pH | 6.3 (5.8-6.6) | 6.2 (5.6-6.5) | 6.2 (5.8-6.5) | 6.1 (5.8-6.6) |

***Abbreviations:*** CTRL, control; MDD, major depressive disorder; BPD, bipolar disorder; FST, freezer storage time; SCZ, schizophrenia; PMI, postmortem interval. ^*^Unless otherwise indicated

*Postmortem brain Cohort 5*

The NSW Brain Tissue Resource Centre cohort (Cohort 5, Supplementary Table 5) consists of a total of 36 cases with schizophrenia, and 33 matched controls. Tissues were dissected from the orbitofrontal cortex (BA11) from the second and third slices coronally sectioned from the fresh hemisphere (each slice was 8-10 mm thick). 45 individuals were male and 24 female, and samples matched by age, PMI and RIN. Postmortem brain tissue was collected at the New South Wales Brain Tissue Resource Centre at the University of Sydney which is supported by the University of Sydney. Research reported in this publication was supported by the National Institute of Alcohol Abuse and Alcoholism of the National Institutes of Health under Award Number NIAAA012725-15. Informed consent was given by all donors or their next of kin for brain autopsy. Groups were matched according to psychiatric diagnoses, postmortem interval (PMI), age at death, and RNA integrity number (RIN) (Supplementary Table 5).

**Supplementary Table 5**. Demographics from NSW Brain Tissue Resource Centre postmortem cohort with samples from the orbitofrontal cortex (BA11).

|  | **Group (mean±standard deviation*)** | |
| --- | --- | --- |
| **Descriptive** | **Control** | **SZ** |
| Subjects | 33 | 36 |
| Age at death (years) | 57±4.32 | 41.69±4.76 |
| Sex (male:female) | 17:0 | 17:0 |
| Cause of death | Natural death (11), accident (6) | Suicide (17) |
| Brain pH | 6.49±0.06 | 6.60±0.07 |
| PMI (hours) | 34.01±4.94 | 41.69±4.76 |
| RIN | 6.16 | 6.47 |

***Abbreviations:*** PMI, postmortem interval; SZ, schizophrenia; RIN, RNA integrity number. ^*^Unless otherwise indicated

*Postmortem brain Cohort 6*

The Douglas-Bell Canada Brain Bank cohort (Cohort 6, Supplementary Table 6) consists of a total of 17 cases with major depressive disorder who died by suicide, and 17 matched controls. Tissues from BA9 were dissected from the lateral part of the superior frontal gyrus. Cases were all males, and matched by age, PMI and RIN. Cause of death was determined by the Quebec Coroner’s office, and frozen grey matter from BA9 isolated by trained neuroanatomists. Psychological autopsies were performed using proxy-based interviews. Cases met criteria for MDD and died by suicide, and controls died suddenly and did not have evidence of any axis I disorders. The study was approved by the Douglas Hospital Research Ethics Board and written informed consent was received from the next of kin.

**Supplementary Table 6**. Demographics from Douglas-Bell Canada Brain Bank postmortem cohort with samples from the dorsolateral prefrontal cortex (BA9).

|  | **Group (mean±standard deviation*)** | |
| --- | --- | --- |
| **Descriptive** | **Control** | **MDD** |
| Subjects | 17 | 17 |
| Age at death (years) | 38±4.32 | 41.69±4.76 |
| Sex (male:female) | 17:0 | 17:0 |
| Cause of death | Natural death (11), accident (6) | Suicide (17) |
| Brain pH | 6.49±0.06 | 6.60±0.07 |
| PMI (hours) | 34.01±4.94 | 41.69±4.76 |
| RIN | 6.16 | 6.47 |

***Abbreviations:*** MDD, major depressive disorder; PMI, postmortem interval; RIN, RNA integrity number. ^*^Unless otherwise indicated

**2. Primer design for real-time quantitative PCR experiments**

**Supplementary Table 7.** RT-qPCR primer design

|  | **Details** |  |  |  |  | | **FKBP5 target** | **Region** |
| --- | --- | --- | --- | --- | --- | --- | --- | --- |
| 1 | IDT Ref | Hs.PT.58.813038 (all transcripts Exon 11-12) | | | |  | All transcripts | Exon 11-12 |
|  | Probe | /56-FAM/CTG TTG AAT /ZEN/GCT GTG ACA AGG CCC /3IABkFQ/ | | | | |  |  |
|  | Primer 1 | ATG TGC TAC CTG AAG CTT AGA G | |  | |  |  |  |
|  | Primer 2 | CCC TCC TAT ACA AGC CTT TCT C | |  | |  |  |  |
| 2 | IDT Ref | Hs.PT.58.20523859 (all transcripts Exon 5-6) | | | |  | All transcripts | Exon 5-6 |
|  | Probe | /56-FAM/AGAGATATG/ZEN/CCATTTACTGTGCAAACCAGA/3IABkFQ/ | | | | |  |  |
|  | Primer 1 | GAACCATTTGTCTTTAGTCTTGGC | |  | |  |  |  |
|  | Primer 2 | CGAGGGAATTTTAGGGAGACTG | |  | |  |  |  |
| 3 | IDT Ref | Hs.PT.39a.22214836 (GAPDH (NM_002046) Exon 2-3) | | | |  | GAPDH | Exon 2-3 |
|  | Probe | /56-FAM/AAGGTCGGA/ZEN/GTCAACGGATTTGGTC/3IABkFQ/ | | | | |  |  |
|  | Primer 1 | ACATCGCTCAGACACCATG | |  | |  |  |  |
|  | Primer 2 | TGTAGTTGAGGTCAATGAAGGG | |  | |  |  |  |
| 4 | IDT Ref | Hs.PT.39a.22214847 (ACTB Exon 1-2) | |  | |  | ACTB | Exon 1-2 |
|  | Probe |  |  |  | |  |  |  |
|  | Primer 1 |  |  |  | |  |  |  |
|  | Primer 2 |  |  |  | |  |  |  |

**3. Antibody validation**

To validate the FKBP51 antibodies used in our study, immunoblots were performed in FKBP51 knock out (KO) cells. The KO cells were generated from the SH-SY5Y human neuroblastoma cell line using CRISPR-Cas9 (Martinelli et al.). Considering FKBP51 is lowly expressed at baseline, the synthetic glucocorticoid receptor agonist dexamethasone was also added to induced FKBP5 expression and increase visible expression of FKBP51 protein (Figure S1). Protein was extracted from cell lysates and 20ug was loaded for immunoblot analyses. FKBP51 antibodies (Supplementary Table 5) were applied (on individually run membranes) at 1:1000, and membranes were imaged according to the general methods.

**Supplementary Table 8.** FKBP51 antibodies validated in FKBP51 knock-out cells

| Name | Type | Concentration | Number | Antibody RRID | Company |
| --- | --- | --- | --- | --- | --- |
| Rabbit anti-FKBP51 | polyclonal | 1:1000 | #8245S | AB_10831198 | Cell Signaling, Danvers, MA, USA |
| Rabbit anti-FKBP51 | polyclonal | 1:1000 | #A301-429A | AB_961003 | Bethyl Laboratories, Montgomery, TX, USA |
| Mouse anti-FKBP51 | polyclonal | 1:1000 | # sc-271547 | AB_10649040 | Santa Cruz Biotechnology, Dallas, TX, USA |

**
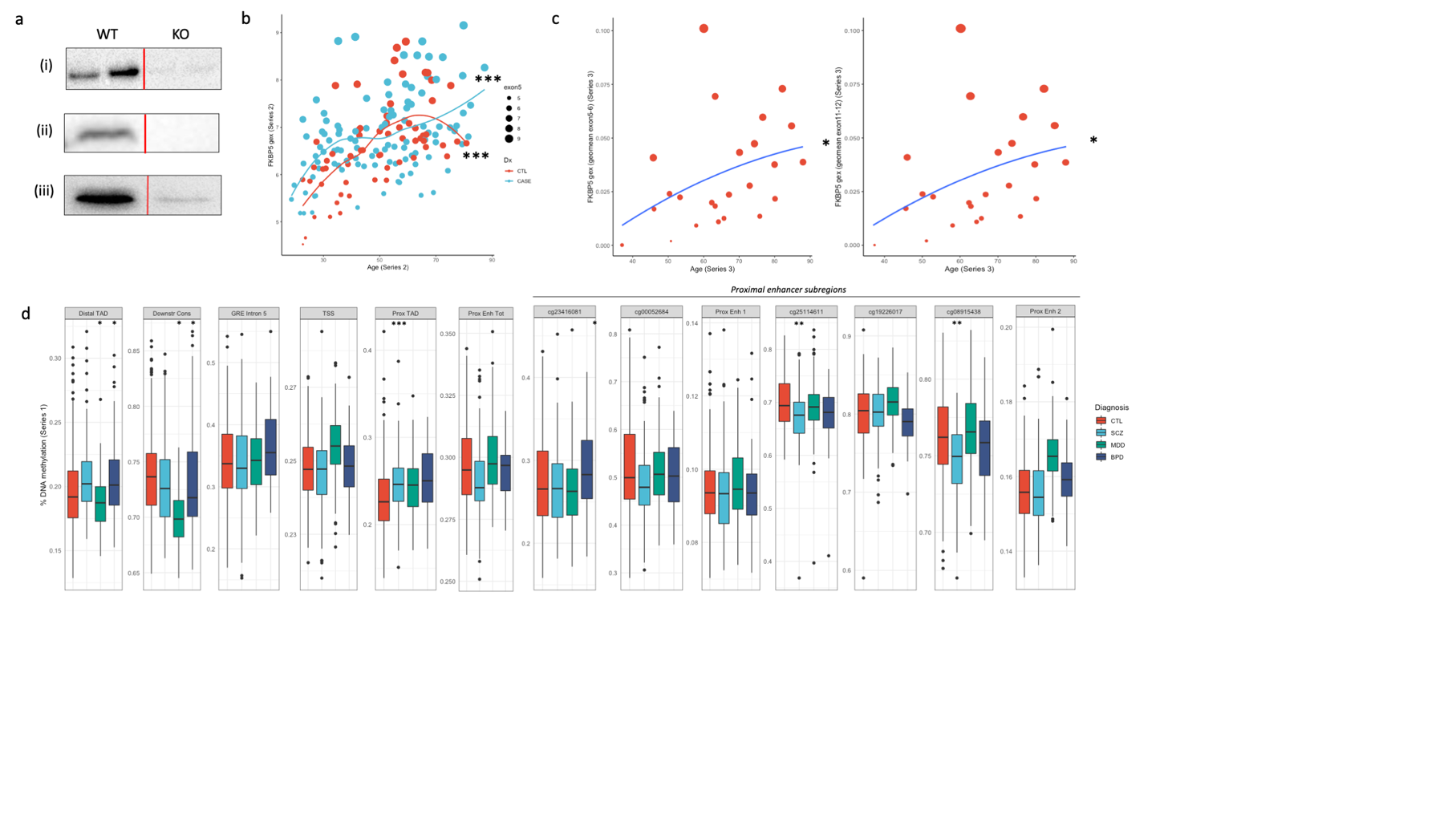
**

**Supplementary Figure 1.** Validation of FKBP51 antibodies in *FKBP5* knock-out HELA cells using immunoblot. All FKBP51 antibodies ([i] Cell Signalling #8245S, [ii] Bethyl #A301-429A, [iii] Santa Cruz #D-4) showed specific expression in wildtype (WT) cells but not *FKBP5* knock-out (KO) cells, indicating their specificity.

**Supplementary Table 9**. Summary of antibodies used for immunohistochemistry experiments

|  | Antibody | Concentration | Catalog Number | RRID | Supplier; Country |
| --- | --- | --- | --- | --- | --- |
| Primary Abs | Rabbit anti-FKBP5 | 1:200 | #8245S | AB_10831198 | Cell Signalling Technologies; MA, USA |
|  | Goat anti-GAD1 | 1:26 | #AF2086 | AB_2107724 | R&D Systems; MN, USA |
|  | Mouse anti-NeuN | 1:100 | #MAB377 | AB_2298772 | Sigma-Aldrich; MA, USA |
|  | Mouse anti-FKBP5 | 1:200 | #sc-271547 | AB_10649040 | Santa Cruz; TX, USA |
|  | Rabbit anti-TMEM119 | 1:100 | #ab185333 | AB_2687894 | Abcam, Cambridge UK |
|  | Chicken anti-GFAP | 1:500 | #AB5541 | AB_177521 | Sigma-Aldrich; MA, USA |
| Secondary Abs | Donkey anti-rabbit 488 | 1:500 | #A32790 | AB_2762833 | Invitrogen; MA, USA |
|  | Donkey anti-goat 594 | 1:500 | #A32758 | AB_2762828 | Invitrogen; MA, USA |
|  | Donkey anti-mouse 647 | 1:500 | #A32787 | AB_2762830 | Invitrogen; MA, USA |
|  | Donkey anti-chicken 647 | 1:300 | #703-605-155 | AB_2340379 | Jackson Immuno Research; PA, USA |

**4. Fluorescence immunohistochemistry method and quantification**

*Triple-label fluorescence immunohistochemistry staining method*

Triple-label fluorescence immunohistochemistry was performed to explore the cell-type specific localisation patterns of the FKBP51 protein. Fresh-frozen tissue blocks ~1.5cm × 1.5cm were cryosectioned at 20um (Cohort 3) and 14µm (Cohort 4), mounted on SuperfrostTM Plus Gold slides, and stored at -80oC until required. For this study, two tissue sections per subject were used. Sections were thawed at 37ºC and post-fixed with 4% paraformaldehyde for 10 mins. Antigen retrieval was performed using a solution of 0.05% Tween-20 with 10mM citric acid buffer. Tissue samples were submerged in the buffer then heated in a microwave at 600 Watts for 60 secs, then 960 Watts for 20 seconds, followed by 120 Watts for 10 mins. For both cohorts, tissue was permeabilised with 0.3% Triton X-100 in phosphate buffered saline (PBS) for 5 mins. Non-specific binding was blocked using a solution of 10% normal donkey serum in blocking buffer (1% w/v bovine serum albumin in a solution of PBS containing 0.3M glycine) for 1hr at 37ºC. Sections were then incubated with the primary antibodies suspended in blocking solution overnight (16hrs) at 4ºC, and subsequently washed with 0.1% Tween-20 in PBS. Secondary antibodies were diluted in blocking solution, and then applied in the dark, at room temperature for 1 hr. Sections were washed with 0.1% Tween-20 in PBS, then incubated with DAPI/HOECHST 33342 nuclear marker (1:1000, Thermo Fischer Scientific (MA, USA), catalog #H3570) in blocking solution at room temperature for 5mins. Finally, sections were washed with 0.1% Tween- 20 in PBS, 50% ethanol for 1min and 70% ethanol for 1min, then counterstained with Autofluorescence Eliminator Reagent (Merck Millipore (MA, USA), catalog #2160) at room temperature for 5mins. 70% ethanol was used to wash the counterstain off prior to sealing. Tissue samples were cover-slipped using Fluoromount-GTM Mounting Medium (Thermo Fisher Scientific (MA, USA), catalog #00-4958-02) and sealed with generic clear nail polish. The sealed slides were stored in the dark at 4ºC until imaged.

*Imaging*

DMi8 THUNDER Inverted 3d Live Cell Fluorescence Microscope

Merged tile scan images were collected for the entirety of each tissue section (two per subject) using the HC PL APO 20x/ 0.80 PH2 objective of a DMi8 THUNDER Fluorescence Microscope equipped with a Leica DFC9000 GTC (4.2 MP sCMOS) camera. Excitation of fluorophores was induced by the integrated LED light source, with individual wavelengths separated by the quad cube filter set allowing isolation of DAPI (405nm), FITC (488nm), TRITC (594nm) and Cy5 (647nm). Once the outlines of the tissue section was set, Adaptive Focus Control was optimised for the 647nm channel and set to adjust the focus of each image within the tilescan (~250-300 images/ tilescan), with a pixel size of 6.5um. Once captured, the tilescan images were merged and Large Volume Computational Clearing (LVCC) applied which considers all optical parameters, producing a haze-free image with a better contrast and resolution for analysis (Schumacher and Bertrand 2019). All microscope settings including exposure time (ms), signal intensity and emission filters were kept constant across samples.

SP8 Confocal Microscope

Z-stack images were captured using the HC PL APO 40x/ 1.30 OIL CS2 objective of a Leica SP8 Confocal Microscope. Excitation of fluorophores was induced by the integrated 405nm, 488nm, 552nm and 638nm lasers. For each sample, six z-stack images with an average of twelve z-sections (step size of 1um, resolution of 512x512 pixels initially, followed by images at 2048 x 2048 imaged through the whole thickness of the mounted tissue) were semi-randomly acquired within the superficial (~LI-III) and deep (~LV/V-VI) layers of the grey cortical matter (three of each). Semi-random acquisition refers to similar pre-chosen locations on the boundary of the grey or white matter, for the superficial and deep layers respectively. During imaging, brief analysis of the gyri and cellular structure confirmed consistent image location across samples. The SP8 is equipped with a sCMOS Hamamatsu W-View Gemini camera, capable of up to 2000 x 1000 pixels per image. All microscope settings including laser power, gain and exposure were corrected for across samples.

*Quantification and analysis*

Thunder images

Analysis of the cortical distribution of FKBP51 through the layers were performed on the two LVCC Merged DMi8 THUNDER tilescans for each subject. The main focus of this analysis was to determine if any differences, whether morphological or density-related, were apparent in FKBP51 expression throughout the cortical layers. On each of the two tile scans per subject, using the Leica LasX Software, two independent cropped images were taken at random from the larger LVCC merged tilescan. Regions were 5x1mm in size and chosen based on the extent to which they spanned the entire cortical grey matter, specifically from the external plexiform layer of the grey matter through to the superficial layers of the underlaying white matter. QuPath (Version 0.3.2) (Bankhead, Loughrey et al. 2017) was used to open the images, and by toggling between the NeuN and FKBP51 channels, NeuN-positive (NeuN+) stains which show a layer specific morphology were used to determine the bounds of each cortical layer, against which the FKBP51 stain profile was compared.

For quantitative analysis, the cropped images were loaded into QuPath (Version 0.3.2), where the *rectangle region of interest* (ROI) tool was used to select six smaller (400x400um) ROIs, two for ~LI-II, two for ~LV-VI and two for the superficial layers of the underlying white matter (microglia only). The *cell detection* tool was used to highlight all the individual NeuN+ detections within the defined bounds of each of the six ROIs, and by toggling between channels, manual observation was used to determine the number of NeuN+ detections positive for FKBP51 (FKBP51+). This information was then used to calculate the percentage of NeuN+ detections which were FKBP51+.

Confocal images

For cellular quantification of the confocal images, the six Z-stacks for each subject were imported into ImageJ with the Fiji plugin (Schindelin, Arganda-Carreras et al. 2012), and merged using the Z project tool (maximum intensity projection). The max projection images were imported into QuPath (Version 0.3.2), where the cell detection and show detection measurements tools were utilised to quantify staining. Neuronal data was collected on a single-cell basis with the quantity, area and intensity of individual NeuN+ stains recorded, including the intensity of HOECHST 33342 and FKBP51 staining within the bounds of each NeuN stained neuron. Finally, the average intensity of FKBP51 staining was determined per image for each FKBP51 detection.

**5. Golgi-Cox staining method and quantification**

*Golgi-Cox staining method*

Blocks of fresh frozen tissue (~1 cm^3^) from the orbitofrontal cortex (Brodmann’s Area 11) were stained using the FD Rapid Golgistain^TM^ Kit (FD Neurotechnologies, Columbia, MD, USA). Briefly, tissue blocks were immersed in 5 mL of impregnation solution containing a 1:1 mixture of provided Solution A (potassium dichromate and mercuric chloride [proprietary concentrations]) and Solution B (potassium chromate [proprietary concentration]) and then stored in the dark at room temperature (RT) for 6 hours. The impregnation solution was replaced after this time and blocks were returned to storage in the dark at RT for 2 weeks. Tissue blocks were transferred to Solution C (proprietary solution) for cryoprotection and stored at RT in the dark for 7 days, replacing Solution C every 24 hours. Blocks were taken out of Solution C and were frozen slowly in iso-pentane cooled to at least -70 °C in dry ice and kept at -80 °C until sectioned.

Coronal sections (150 µm) of the frozen blocks were cut using a Lecia CM1950 Cryostat (Leica Biosystems, Wetzlar, Germany) with an internal chamber temperature of -21 °C, using a blade cooled with dry ice to prevent tissue from sticking to the blade. Sections were transferred onto gelatin-coated microscope slides (FD Neurotechnologies) covered with Solution C to prevent damage from dry thawing. Excess Solution C was absorbed with filter paper so that sections remained stationary on the slide. The sections were then completely dried, fixing sections to slides, at RT in a vacuum desiccator (24 - 48 hours). Sections were then rehydrated on slides in excess Milli-Q water for 10 minutes. They were then immersed in a proprietary ammonia-based developing solution for 10 minutes, followed by an 8 minute wash in water, following the manufacturer’s instructions. Sections were gradually dehydrated of developing solution by sequential 4 minute immersions in increasing concentrations of ethanol (50 %, 75 %, 95 %, and four times in 100 %). Sections were cleared three times in xylene, covered with coverslips, and sealed with Permount^®^ (Fisher Scientific, Waltham, MA, USA). Slides were left to completely set for one week and then cleaned of excess Permount^®^ with 70 % ethanol.

*Microscopy sampling of dendrites*

All data collection was collected blind to group and matched with individuals after data collection and prior to statistical analysis. Regular stratified layers of cells were clearly identified. Layers (I-VI) were defined on slides according to the morphological features outlined by von Economo and Koskinas (2008). Pyramidal neurons are classically found in layers II, III and V and were as such selected as regions for analysis (von Economo and Koskinas 2008). Layers II and III were difficult to distinguish under the Golgi-Cox stain and thus grouped for analysis.

A fractionator sampling strategy was adapted from West (1993) to minimise bias in selection of regions. Three to six randomly sampled pyramidal neurons from randomised coordinates were selected within each defined subregion at 20x magnification using bright field imaging on a Leica DMi8 microscope (Leica Microsystems, ‎Wetzlar‎, Germany). Neurons were assessed for quality of staining and dendritic arborage. For each section, an adapted optical fractionator method was used to randomly select coordinates within each layer. This was repeated until 3-6 pyramidal neurons were imaged. Neurons with poor staining quality were rejected and new neurons selected. *Z*-stacks of selected neurons, centred on apical dendrites, were collected at 63x magnification in oil using a 0.6 μm step size. Settings for exposure and gain were optimised and kept as constant as possible between samples to ensure consistent imaging at 2048 x 2048 pixels.

*Golgi analysis*

Dendrite sampling was conducted as outlined in previous studies (Radley, Rocher et al. 2005, Anderson, Glanz et al. 2016). Selection criteria for dendrites required that segments were: less than 3 µm in diameter (as larger segments in pyramidal neurons have higher spine variability); within 100 µm of the coronal surface of the section to avoid any optical limitations of the microscope, relatively parallel to the coronal surface of the section to avoid distortion that can arise from thick z-stack imaging; separate from other dendritic segments to avoid any errors in modelling and spine analysis, and at least 20 µm in length. Dendrites were analysed using the open source program ImageJ with the Fiji plugin (Schindelin, Arganda-Carreras et al. 2012). Briefly, the dendrite was traced to determine segment length and distance from the soma. Along the apical dendrite of each accepted neuron, 3-6 randomly selected segments that met these criteria were analysed. All dendritic spines located along the segment within the same x-y plane as the segment were counted, measured manually and morphologically categorised into filopodia, thin, stubby and mushroom spines as outlined previously by Risher, Ustunkaya et al. (2014).

| **B. EXTENDED RESULTS**  **Supplementary Table 10.** Summary of the research questions and statistical methods applied in this study. | | | | | | |
| --- | --- | --- | --- | --- | --- | --- |
| **Research question** | **Statistical method** | **Statistical**  **model** | **Covariates (only selected if significant in the model)** | **Cohort and sample size** | **Experimental Method** | **Result** |
| Does *FKBP5* gene expression significantly differ between cases (grouped and independent diagnosis) and controls? | Linear model | lm(gex~cov+diagnosis) | Age, sex, PMI, pH, qSVA†, cell type* | Cohort 1 (14 years+ only)  n=180 controls  n=121 schizophrenia  n=144 major depression  n=63 bipolar | Bulk RNA sequencing | *FKBP5* expression levels were significantly heightened in cases, driven by schizophrenia subjects. |
|  |  |  | Age, sex, PMI, pH, suicide, RIN, type of death, experimental batch variables | Cohort 2  n=62 controls  n=68 schizophrenia  n=24 major depression  n=15 bipolar | Microarray |  |
| Does FKBP51 protein expression significantly differ between cases (grouped and independent diagnosis) and controls? | Linear model | lm(gex~cov+diagnosis) | Age, sex, PMI, pH, suicide, RIN, type of death | Cohort 2  n=20 controls  n=20 schizophrenia  n=20 major depression  n=16 bipolar disorder | Western blot | FKBP51 expression levels were significantly heightened in cases, driven by schizophrenia subjects. |
|  |  |  | Age, sex, PMI, pH | Cohort 4  n=15 controls  n=15 schizophrenia  n=15 major depression  n=15 bipolar disorder |  |  |
| Do *FKBP5* gene expression and FKBP51 protein expression correlate? | Spearman’s correlation | cor(gex,protein expression) | - | Cohort 2  n=20 controls  n=20 schizophrenia  n=20 major depression  n=16 bipolar disorder | Bulk RNA sequencing and western blot | There is a a strong, positive correlation between *FKBP5* gex and FKBP51 protein levels across all subjects. |
| Is there an additive effect of the rs1360780 risk genotype on case status causing further increased *FKBP5* gene expression? | Linear model | lm(gex~cov+SNP+diagnosis) | Age, sex, PMI, pH, qSVA†, cell type* | Cohort 1 (14 years+ only)  n=108/64 T/C controls  n=69/45 T/C schizophrenia  n=78/62 T/C major depression  n=32/28 T/C bipolar | Bulk RNA sequencing and SNP genotyping | No additive effect of SNP risk genotype is present. |
| Are *FKBP5* gene expression levels correlated with age? | Spearman’s correlation | cor(gex, age) | - | Cohort 1 (14 years+ only)  n=180 controls | Bulk RNA sequencing | Strong correlation of *FKBP5* gene expression with age in neurotypical controls. |
|  |  |  |  | Cohort 2  n=62 controls | Microarray |  |
|  |  |  |  | Cohort 3  n=24 controls | qPCR (two probes) |  |
| Are FKBP51 protein expression levels correlated with age? | Spearman’s correlation | cor(protein expression, age) | - | Cohort 2  n=20 controls | Western blot | Strong correlation of FKBP51 protein with age in neurotypical controls. |
|  |  |  |  | Cohort 3  n=24 controls |  |  |
|  |  |  |  | Cohort 4  n=15 controls |  |  |
| Are the effects of aging on *FKBP5* gene expression further increased in psychiatric disorders? | sm.ancova | sm.ancova(gex~cov+diagnosis) | Age, sex, race, RIN, PMI, qSVA, cell type* | Cohort 1 (14 years+ only)  n=180 controls  n=121 schizophrenia  n=144 major depression  n=63 bipolar | Bulk RNA sequencing | In Cohort 1, the *FKBP5* ageing trajectory was significantly heightened in schizophrenia subjects compared to controls.  In Cohort 2, a similar trend was seen but it did not reach statistical significance (*P*=0.056) |
|  |  |  | Age, sex, PMI, pH, suicide, RIN, type of death, experimental batch variables | Cohort 2  n=62 controls  n=68 schizophrenia  n=24 major depression  n=15 bipolar | Microarray |  |
| Do the case-control differences in *FKBP5* gene expression vary according to cell-type? | Linear model | lm(gex~cov+diagnosis) | Age, PMI, pH, RIN, library prep batch, sequencing batch | Cohort 5  n=33 controls  n=36 schizophrenia | Single-nucleus RNA sequencing  Comparison in each major cell-type cluster and excitatory neuron subcluster based on cortical layer location. | In schizophrenia, *FKBP5* gene expression was increased in all cell types in the major cell-type clusters except interneurons, with strong effects seen in excitatory cell types. In the excitatory neuron cortical-layer subclusters, *FKBP5* gene expression was higher only in the supragranular layer.  In depression, the only case-control difference in *FKBP5* geneexpression was found in the excitatory neuron group and supragranular neurons specifically but neither survived correction for multiple cell-type comparisons. |
|  |  |  | Age, PMI, pH, RIN, sequencing batch | Cohort 6  n=17 controls  n=17 depression |  |  |
| Is there an increase in FKBP51 protein expression on superficial layer excitatory neurons in schizophrenia? | Linear model | lm(protein expression~cov+diagnosis) | Age, sex, PMI, brain weight, pH, freezer storage time, subject | Cohort 4  n=15 controls  n=15 schizophrenia  n=15 major depression  n=15 bipolar disorder  To improve power, analyses were performed on count and single-cell staining intensity data for >12,000 NeuN+ neurons. | Immunohistochemistry | In schizophrenia, FKBP51 staining intensity was higher in the supgranular layer of the cortex but not the deep layer. In depression, FKBP51 staining intensity was lower in the supgranular layer of the cortex but not the deep layer. There was no differences seen in bipolar disorder. |
| Are *FKBP5*/1 mRNA/protein expression levels correlated with age specifically in superficial layer neurons? | Spearman’s correlation | cor(gex, age)  cor(protein expression, age) | - | Cohort 5 (cases and controls combined)  n=33 controls  n=36 schizophrenia | Single-nucleus RNA sequencing | Age was correlated with *FKBP5* gene expression in all the major cell-type clusters. The most striking correlation was in the excitatory neuron cluster. In the excitatory neuron subclusters, the strongest aging effect was in the supragranular excitatory neurons. |
|  |  |  |  | Cohort 6 (cases and controls combined)  n=17 controls  n=17 depression | Single-nucleus RNA sequencing | Age was strongly correlated with *FKBP5* gene expression in the excitatory neuron cluster. The aging effect was strongest in, and specific to, cortical layer 2-4 excitatory neurons. |
|  |  |  |  | Cohort 3  n=24 controls | RNAscope | Positive correlation of age and *FKBP5* transcript dots in the superficial layers but not the deep layers. |
|  |  |  |  | Cohort 3  n=24 controls | Immunohistochemistry | Positive correlation of age on FKBP51 protein expression in NeuN+ neurons was also specific to the superficial but not deep cortical layers. |
| Are the age effects on *FKBP5*/1 mRNA/protein expression levels in superficial layer neurons pronounced in cases? | Spearman’s correlation | cor(protein expression, age) | - | Cohort 4  n=15 controls  n=15 schizophrenia  n=15 major depression  n=15 bipolar disorder  To improve power, analyses were performed on count and single-cell staining intensity data for >12,000 NeuN+ neurons. | Immunohistochemistry | Schizophrenia subjects showed the strongest correlation of age on the number of FKBP51+ neurons specific to the superficial but not deep cortical layers. MDD and BP subjects showed a weak positive correlation of age and number of FKBP51+ cells only in the deep layers.  Schizophrenia subjects showed the strongest positive correlation of FKBP51+ neuronal staining intensity with age, in both the superficial and deep layers.  In MDD, FKBP51+ neuronal staining intensity was positively correlated with age only in the deep layers. In BP, the effects of age were present in both the superficial and deep layers.  In controls, there was no correlation of age on the numbers of FKBP51+ neurons or neuronal staining intensity in neither the deep nor superficial layers |
| Is elevated superficial neuron *FKBP5* gene expression levels in schizophrenia correlated with dendritic spine architecture (mushroom, stubby, filopodia and thin spines)? | Partial correlation | pcor(gex, spine density+cov)  pcor(gex 1, gex 2+cov) | Age, sex, PMI | Cohort 5  n=8 control  n=11 schizophrenia | Single-nucleus RNA sequencing  Golgi-Cox staining | *FKBP5* gene expression levels were specifically, highly and negatively correlated with mushroom spine density.  *BDNF* gene expression levels were strongly and inversely correlated with *FKBP5* gene expression levels and strongly and specifically correlated with mushroom spines. |

* To account for potential confounding of different cell types, we used cell type variables derived from the RNA deconvolution model previously described (Darmanis, Sloan et al. 2015)

† To remove residual confounding by RNA degradation, we used the quality surrogate variable analysis (qSVA) framework previously described (Jaffe, Tao et al. 2017)

**Supplementary Table 11.** Sample sizes for genotype analyses

| **Rs1360780,** Sample size (n) | | |
| --- | --- | --- |
|  | T carrier | CC |
| Control | 108 | 64 |
| Schizophrenia | 69 | 45 |
| Major Depression | 78 | 62 |
| Bipolar Disorder | 32 | 28 |

**
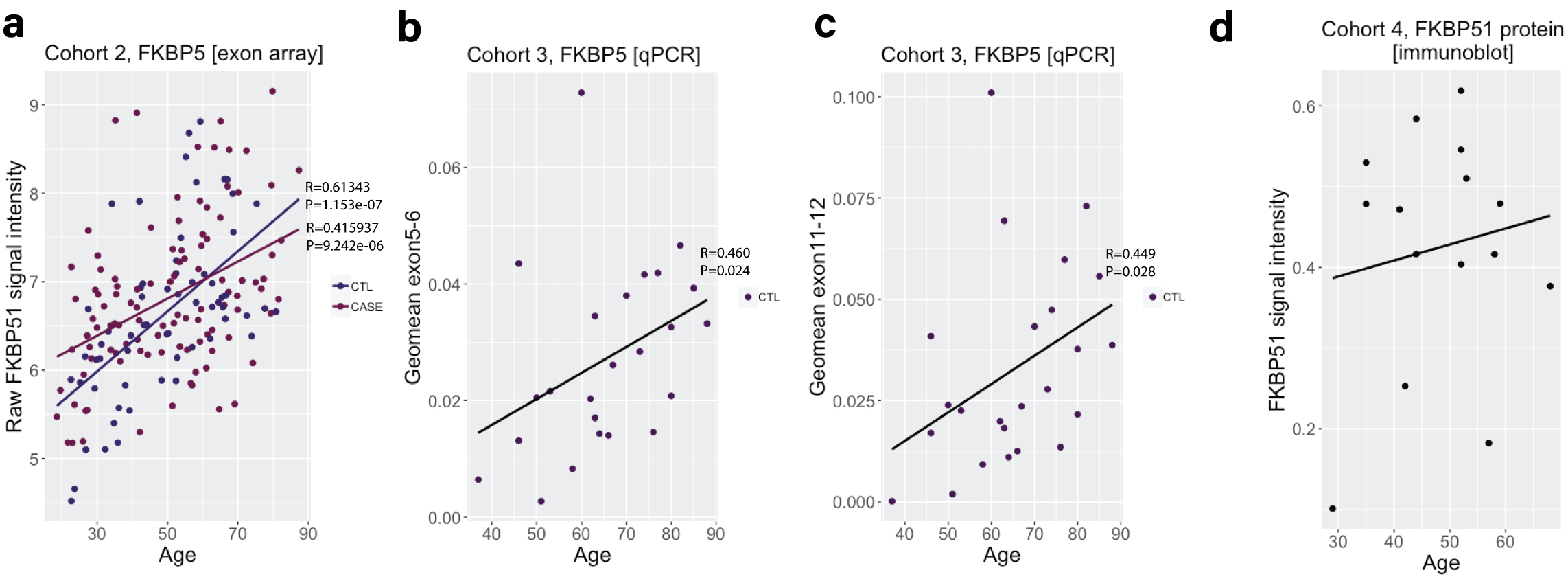
**

**Supplementary Figure 2. Association of *FKBP5/*1 expression with age in Cohort 2, 3 and 4. (a)** Association of *FKBP5* gene expression with age in Cohort 2. *FKBP5* was positively and significantly associated with age in both cases and controls (CTL), with heightened expression at older ages in cases vs controls. The difference in case vs control *FKBP5* aging trajectory, measured using sm.ancova, did not reach statistical significance (*P*=0.056). **(b-c)** Results of quantitative PCR experiments assessing the correlation between *FKBP5* expression with age in Cohort 3. *FKBP5* was significantly associated with age with two independent probes targeting all transcripts either at (**b**) *FKBP5* exon 5-6 or (**c**)11-12. Cohort refers to the cohorts used in each analysis, as detailed in Table 1. **(d)** Association of FKBP51 protein expression with age in Cohort 4. *FKBP5* was not correlated with age, although it is important to note that there were few subjects over 60 in this sample (n=1).

**Supplementary Table 12.** Schizophrenia vs control differences in *FKBP5* gene expression between the major cell clusters and excitatory neuron sub-clusters from Cohort 5, using linear regression modelling. Results of the coefficients for diagnoses derived from the linear regressions (*lm* function) are presented. Nominal and FDR-corrected P values for multiple comparisons across cell types are reported.

| **COHORT 5**  **Major/sub-cluster name** | **Cortical layer(s)** | **t-value** | **P­_nom_** | **P_FDR_** |
| --- | --- | --- | --- | --- |
| Oligodendrocytes | - | 3.128 | 0.002701 | **0.006302333** |
| Astrocytes | - | 3.135 | 0.002644 | **0.006302333** |
| Oligodendrocyte progenitor cell | - | 3.452 | 0.00102 | **0.006302333** |
| Inhibitory neurons | - | 1.937 | 0.05740 | 0.057400000 |
| Endothelial cells | - | 2.745 | 0.00794 | **0.011116000** |
| Microglia | - | 2.578 | 0.012354 | **0.014413000** |
| Excitatory | - | 2.969 | 0.00426 | **0.007455000** |
| *Posthoc:* |  |  |  |  |
| Ex 2 | 2-3 | **3.256** | **0.00186** | **0.00930000** |
| Ex 3 | 3-5 | 2.123 | 0.03793 | 0.06321667 |
| Ex 4 | 4-6 | 2.265 | 0.02721 | 0.06321667 |
| Ex 5 | 4-6 | 1.956 | 0.0551 | 0.06887500 |
| Ex 6 | 5-6 | 1.410 | 0.163877 | 0.16387700 |

**Supplementary Table 13.** Depression vs control differences in *FKBP5* gene expression between excitatory neuron sub-clusters from Cohort 6 using linear regression modelling. Results of the coefficients for diagnoses derived from the linear regressions (*lm* function) are presented. Nominal and FDR-corrected P values for multiple comparisons across cell types are reported.

| **COHORT 6**  **Major/sub-cluster name** | **Cortical layer(s)** | **t-value** | **P­_nom_** | **P_FDR_** |
| --- | --- | --- | --- | --- |
| Astrocytes | - | 1.079 | 0.29058 | 0.5749333 |
| Endothelium | - | 0.328 | 0.744114 | 0.744114 |
| Microglia | - | 1.335 | 0.18717 | 0.5749333 |
| Oligodendrocytes | - | -0.731 | 0.471 | 0.5749333 |
| Oligodendrocyte progenitor cell | - | 0.766 | 0.4445 | 0.5749333 |
| Inhibitory | - | 0.695 | 0.4928 | 0.5749333 |
| Excitatory | - | **-1.776** | **0.0267** | 0.1869 |
| *Posthoc:* |  |  |  |  |
| Ex 2 | 5 | 0.066 | 0.948 | 0.9480000 |
| Ex 3 | 4-5 | 0.478 | 0.638 | 0.7443333 |
| Ex 4 | 6 | -0.687 | 0.4990 | 0.6986000 |
| Ex 6 | 4-6 | 0.729 | 0.4723 | 0.6986000 |
| Ex 7 | 4-6 | -0.978 | 0.33673 | 0.6986000 |
| Ex 8 | 5-6 | -1.596 | 0.1226 | 0.4291000 |
| Ex 10 | 2-4 | **-2.190** | **0.0364** | 0.2548000 |
|  |  |  |  |  |

**
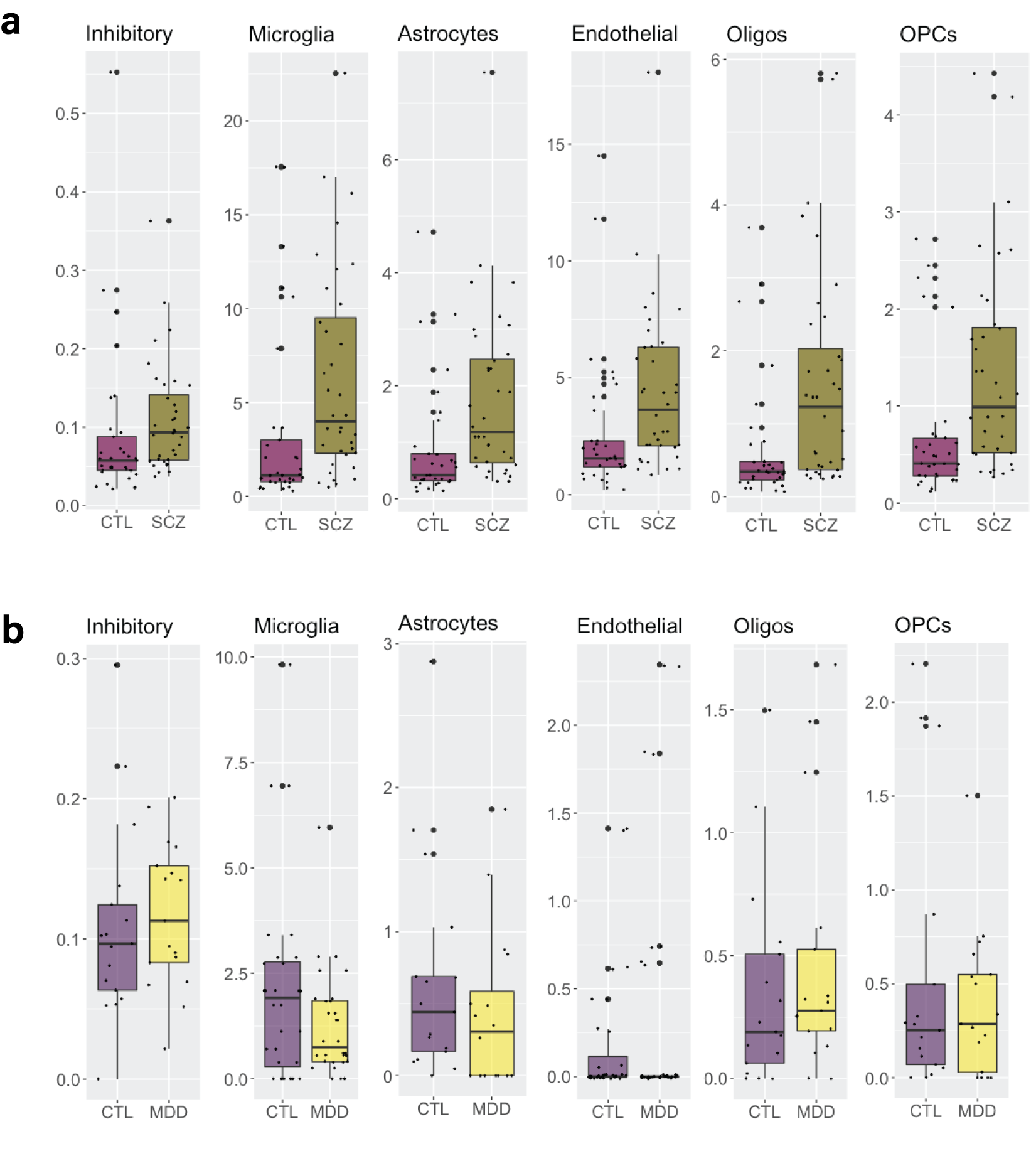
**

**Supplementary Figure 3.** Boxplots showing snRNAseq *FKBP5* gene expression in each individual major cell type cluster (labelled on the top of each plot) from (**a**) Cohort 5 and (**b**) Cohort 6. In schizophrenia, there was a significant difference (P<0.05) in all groups except the inhibitory interneurons (see Supplementary Table 12). In depression, there was no significant difference in *FKBP5* mRNA expression in depression in any of these cell-types. Statistical output is provided in Supplementary Tables 11. Y axis represents mean *FKBP5* gene expression per cluster. *Abbreviations:* CTL, control; Inhibitory, inhibitory neurons, MDD, major depression; oligos, oligodendrocytes; OPCs, oligodendrocyte progenitor cells.

**Supplementary Table 14**. Results of Spearman correlations assessing the relationship between *FKBP5* gene expression with age in each cell cluster from Cohort 5 (schizophrenia and controls combined). Nominal and FDR-corrected P values are reported.

| **Major/sub-cluster name** | **Cortical layer(s)** | **R-value** | **P­_nom_** | **P_FDR_** |
| --- | --- | --- | --- | --- |
| Oligodendrocytes | - | **0.3503878** | **0.003162** | **0.0037180000** |
| Astrocytes | - | **0.3447741** | **0.003718** | **0.0037180000** |
| Oligodendrocyte progenitor cell | - | 0.3747076 | 0.001513 | **0.0035303333** |
| Inhibitory neurons | - | 0.4046593 | 0.0005633 | **0.0019715500** |
| Endothelial cells | - | 0.3533501 | 0.002899 | **0.0037180000** |
| Microglia | - | 0.3478827 | 0.0034 | **0.0037180000** |
| Excitatory neurons | - | **0.5537049** | **8.016e-07** | **0.0000056112** |
| *Posthoc:* |  |  |  |  |
| **Ex 2** | **2-3** | **0.555395** | **7.3e-07** | **0.00000365** |
| **Ex 3** | **3-5** | **0.2683298** | **0.0258** | **0.02580000** |
| **Ex 4** | **4-6** | **0.2930017** | **0.01455** | **0.01818750** |
| **Ex 5** | **4-6** | **0.4972975** | **1.38e-05** | **0.00003450** |
| **Ex 6** | **5-6** | **0.3013647** | **0.01186** | **0.01818750** |

**Supplementary Table 15**. Results of Spearman correlations assessing the relationship between *FKBP5* gene expression with age in each cell cluster from Cohort 6 (depression and controls combined). Nominal and FDR-corrected P values are reported.

| **Major/sub-cluster name** | **Cortical layer(s)** | **R-value** | **P­_nom_** | **P_FDR_** |
| --- | --- | --- | --- | --- |
| Oligodendrocytes | - | **0.4011025** | **0.0187** | **0.0479** |
| Astrocytes | - | **0.4015578** | **0.0205** | **0.0479** |
| Oligodendrocyte progenitor cell | - | 0.3309074 | 0.0559 | 0.0979 |
| Inhibitory neurons | - | 0.2962397 | 0.0889 | 0.1245 |
| Endothelial cells | - | 0.1257452 | 0.4786 | 0.5584 |
| Microglia | - | 0.09823437 | 0.5927 | 0.5927 |
| Excitatory neurons | - | **0.6638643** | **1.87E-05** | **1.31E-04** |
| *Posthoc:* |  |  |  |  |
| Ex 2 | 5 | 0.1197 | 0.5138 | 0.7193 |
| Ex 3 | 4-5 | 0.3377615 | 0.0849 | 0.1827 |
| Ex 4 | 6 | 0.3023 | 0.1044 | 0.1827 |
| Ex 6 | 4-6 | 0.3122614 | 0.0722 | 0.1827 |
| Ex 7 | 4-6 | 0.06969655 | 0.6953 | 0.7267 |
| Ex 8 | 5-6 | 0.06322 | 0.7267 | 0.7267 |
| **Ex 10** | **2-4** | **0.69978** | **4.11E-06** | **2.88E-05** |

**Supplementary Table 16**. Results of Spearman correlations in Cohort 4, assessing the relationship between age and (a) FKBP51 staining intensity on NeuN+ neurons, and (b) the number of FKBP51+ NeuN+ neurons in the ACC.

|  | **Supragranular neurons** | | **Infragranular neurons** | |
| --- | --- | --- | --- | --- |
| **(a)** Age vs FKBP51 staining intensity on NeuN+ neurons | | | | |
|  | R value | P (nominal) value | R value | P (nominal) value |
| All subjects | -0.143087 | 0.2973 | -0.1163699 | 0.3975 |
| Controls | -0.01991194 | 0.9461 | -0.1902697 | 0.5147 |
| Schizophrenia | -0.04410167 | 0.881 | -0.01764067 | 0.9523 |
| Major Depression | -0.2970299 | 0.3024 | -0.2816283 | 0.3293 |
| Bipolar Disorder | -0.4536761 | 0.1194 | 0.008298954 | 0.9785 |
|  |  |  |  |  |
| (b) Age vs the number of FKBP51+ NeuN+ neurons | | | | |
|  | R value | P (nominal) value | R value | P (nominal) value |
| All subjects | **0.2735827** | **0.04327** | 0.06430252 | 0.6409 |
| Controls | 0.4589846 | 0.09876 | 0.2893598 | 0.3157 |
| Schizophrenia | **0.6270845** | **0.01638** | -0.08425741 | 0.7746 |
| Major Depression | -0.1283198 | 0.662 | -0.03311266 | 0.9105 |
| Bipolar Disorder | 0.2402787 | 0.4291 | 0.1448468 | 0.6368 |
|  |  |  |  |  |


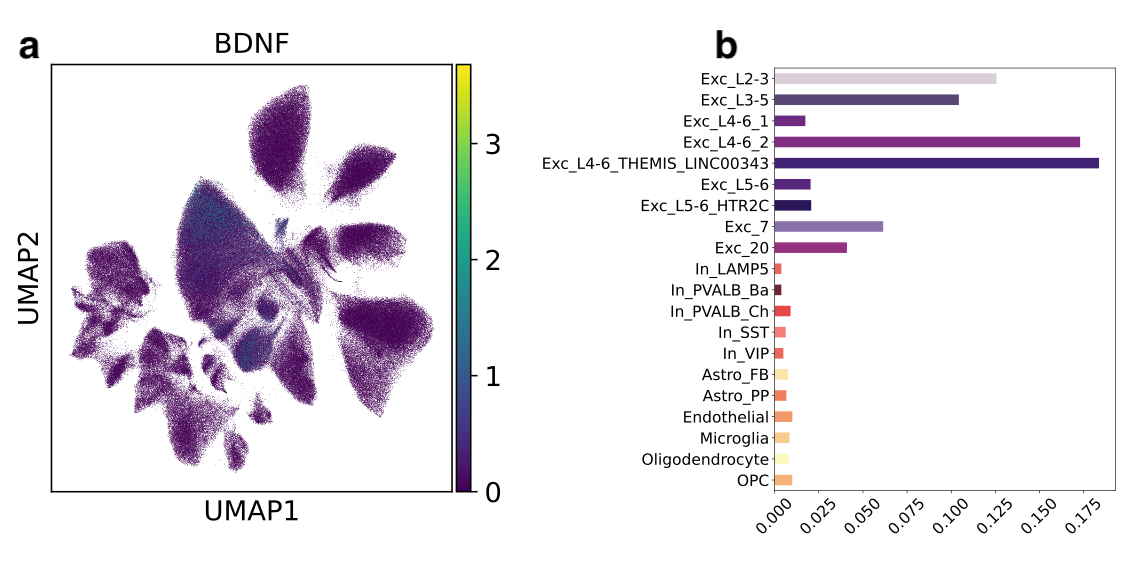


**Supplementary Figure 4.** Dimensionality reduction uniform manifold approximation and projection (UMAP) plots depicting (a) *BDNF* expression across cell clusters and (b) bar plot showing average *FKBP5* gene expression per cell-type cluster from Cohort 5.

C. REFERENCES

- Anderson, R. M., R. M. Glanz, S. B. Johnson, M. M. Miller, S. A. Romig-Martin and J. J. Radley (2016). "Prolonged corticosterone exposure induces dendritic spine remodeling and attrition in the rat medial prefrontal cortex." J Comp Neurol **524**(18): 3729-3746.
- Bankhead, P., M. B. Loughrey, J. A. Fernandez, Y. Dombrowski, D. G. McArt, P. D. Dunne, S. McQuaid, R. T. Gray, L. J. Murray, H. G. Coleman, J. A. James, M. Salto-Tellez and P. W. Hamilton (2017). "QuPath: Open source software for digital pathology image analysis." Sci Rep **7**(1): 16878.
- Darmanis, S., S. A. Sloan, Y. Zhang, M. Enge, C. Caneda, L. M. Shuer, M. G. Hayden Gephart, B. A. Barres and S. R. Quake (2015). "A survey of human brain transcriptome diversity at the single cell level." Proc Natl Acad Sci U S A **112**(23): 7285-7290.
- Jaffe, A. E., R. Tao, A. L. Norris, M. Kealhofer, A. Nellore, J. H. Shin, D. Kim, Y. Jia, T. M. Hyde, J. E. Kleinman, R. E. Straub, J. T. Leek and D. R. Weinberger (2017). "qSVA framework for RNA quality correction in differential expression analysis." Proc Natl Acad Sci U S A **114**(27): 7130-7135.
- Radley, J. J., A. B. Rocher, M. Miller, W. G. M. Janssen, C. Liston, P. R. Hof, B. S. McEwen and J. H. Morrison (2005). "Repeated Stress Induces Dendritic Spine Loss in the Rat Medial Prefrontal Cortex." Cereb Cortex **16**(3): 313-320.
- Risher, W. C., T. Ustunkaya, J. Singh Alvarado and C. Eroglu (2014). "Rapid Golgi analysis method for efficient and unbiased classification of dendritic spines." PLoS One **9**(9): e107591.
- Scarr, E., M. Udawela, E. A. Thomas and B. Dean (2018). "Changed gene expression in subjects with schizophrenia and low cortical muscarinic M1 receptors predicts disrupted upstream pathways interacting with that receptor." Mol Psychiatry **23**(2): 295-303.
- Schindelin, J., I. Arganda-Carreras, E. Frise, V. Kaynig, M. Longair, T. Pietzsch, S. Preibisch, C. Rueden, S. Saalfeld, B. Schmid, J.-Y. Tinevez, D. J. White, V. Hartenstein, K. Eliceiri, P. Tomancak and A. Cardona (2012). "Fiji: an open-source platform for biological-image analysis." Nature Methods **9**: 676.
- Schindelin, J., I. Arganda-Carreras, E. Frise, V. Kaynig, M. Longair, T. Pietzsch, S. Preibisch, C. Rueden, S. Saalfeld, B. Schmid, J. Y. Tinevez, D. J. White, V. Hartenstein, K. Eliceiri, P. Tomancak and A. Cardona (2012). "Fiji: an open-source platform for biological-image analysis." Nat Methods **9**(7): 676-682.
- Schumacher, J. and L. Bertrand (2019). "THUNDER imagers: how do they really work?" THUNDER Imager Technical Note.
- Tao, R., K. N. Davis, C. Li, J. H. Shin, Y. Gao, A. E. Jaffe, M. C. Gondre-Lewis, D. R. Weinberger, J. E. Kleinman and T. M. Hyde (2017). "GAD1 alternative transcripts and DNA methylation in human prefrontal cortex and hippocampus in brain development, schizophrenia." Mol Psychiatry.
- Torrey, E. F., M. Webster, M. Knable, N. Johnston and R. H. Yolken (2000). "The Stanley Foundation brain collection and Neuropathology Consortium." Schizophrenia Research **44**(2): 151-155.
- von Economo, C. and G. N. Koskinas (2008). Atlas of Cytoarchitectonics of the Adult Human Cerebral Cortex. Basil, New York, Karger.
- West, M. J. (1993). "New stereological methods for counting neurons." Neurobiol Aging **14**(4): 275-185.
